# The nature and nurture of education

**DOI:** 10.1038/s41539-018-0023-z

**Published:** 2018-03-23

**Authors:** Pankaj Sah, Michael Fanselow, Gregory J. Quirk, John Hattie, Jason Mattingley, Tracey Tokuhama-Espinosa

**Affiliations:** 1 Queensland Brain Institute, The University of Queensland, Brisbane, QLD 4072 Australia; 2 Staglin Center for Brain and Behavioral Health, University of California, Los Angeles, Los Angeles, CA USA; 3 School of Medicine, University of Puerto Rico, San Juan, Puerto Rico; 4 Graduate School of Education, The University of Melbourne, Parkville, Australia; 5 Applied Educational Research Center, Latin American School of Social Sciences, Harvard Extension School, Harvard University, Cambridge, MA USA

Learning is a life-long endeavour that continues from infancy to old age. We each navigate the learning process in different ways, yielding to our experiences and the circumstances in which we find ourselves. For many people, formal education takes place between the ages of 6–18, where we are educated based on core curricula that are delivered through a schooling system. Many countries have some form of compulsory education for children until they reach adulthood, but the route through the school system can vary greatly. In many first world countries, the choice is between public schools, generally funded by the state, and private schools which are funded through a combination of individual tuition fees, and religious or corporate institutions. School education is generally required by government mandate, and the cost of admission and tuition in public schools is borne by the state. In contrast, entry into a private school is often primarily dependent on socioeconomic status and some form of selection process—either formal testing or religious affiliation.

In many countries, studies have suggested that students attending private schools attain better educational outcomes, and long-term socioeconomic benefits. These data are then used by private schools to encourage attendance in this selective system. Private schools are generally expensive but may provide more individualized programs of study with better student–faculty ratios then those provided by public schools.

The choice parents make in selecting schools for their children, and the differences between public and private schools has been the subject of much debate, that is largely couched in social and economic terms. In a collection of manuscripts published in *npj Science of Learning*, two groups of researchers have approached this discussion from an interesting new direction—genetics. The Nature versus Nurture question has been greatly debated for many years, because it is not entirely clear which is the greatest influence on human development and behaviour. Although we are all born with a specific set of genes, with no control over our genetic allocation, we now know our life-style choices and different experiences though development and maturity also influence gene expression, and thus exert control over our behaviour via epigenetic modifications. Epigenetic mechanisms regulate the structure and activity of the genome in response to cellular and environmental cues, one such mechanism involves DNA methylation. Thus, biological processes are controlled by a combination of inherited genes and the life-long impact of epigenetic modifications that regulates their expression. Who we are is not simply a result of either nature or nurture but rather is shaped by a combination of these factors. Recent advances in genomic and epigenomic sequencing, have led to a growing interest in using this information to predict biological outcomes, and disease pathogenesis and help guide individuals in lifestyle choices and behaviour.

Two papers^1,2^ published in *npj Science of Learning* have worked to address the question of 'Does an individual’s genetic makeup, and epigenetic modification, affect his or her educational attainment?'. Educational attainment is a measure of the highest level of education that an individual has completed at the end of full-time compulsory education. Educational attainment has been shown to strongly correlate with mental and physical health, as well as socioeconomic status, and is one of the strongest predictors of lifetime success, not only economically but also in terms of health and longevity.

In one study,^1^ Smith–Woolley and colleagues looked at educational attainment in three groups of students in Britain that attended either: public schools, private schools or selective schools. The researchers found that as previously reported, students in private schools had higher levels of educational attainment than those in public schools. They then examined the genetic differences between students in these groups, and surprisingly, there were differences in genetic markers between them. Interestingly, when differences in genetics were accounted for, educational attainment differences between students attending the different schools disappeared.

In another study,^2^ van Dongen and colleagues examine the DNA methylation status of genes in people with different levels of educational attainment. They found differential sites of DNA methylation at specific regions (loci) correlate with educational attainment and the methylation status of these sites are largely influenced by environmental factors such as smoking. These sites of differential methylation were found to be located in and near genes with neuronal, immune and developmental functions. Differential levels of DNA methylation in these regions could impact the expression of these genes during critical periods of childhood development. Together, the two studies point to the role of genetics and epigenetic changes in educational outcome. Two accompanying perspective pieces, one by Nick Martin^3^ and the other by Sue Thompson,^4^ provide a commentary on the implications of these studies from the genetic^3^ and educational^4^ viewpoint.

There is a growing interest in genomic and epigenomic sequencing of different populations, with the data generated being incorporated into many different databases. Large-scale projects like the ENCODE (Encyclopedia of DNA Elements) Consortium, which is an international collaboration of research groups funded by the National Human Genome Research Institute (NHGRI), and the British 100,000 genomes project, led by Genomics England, are leading the way in trying to understand how these factors influence biological processes. The two studies published in *npj Science of Learning* raise the question of the use of genomic data to help predict educational outcomes. Just as the management of our health is increasingly being found to be affected by genetic and epigenetic determinants, it may be that individuals progress through the education system based upon these factors as well.
